# Vaccination with the recombinant major outer membrane protein elicits long-term protection in mice against vaginal shedding and infertility following a *Chlamydia muridarum* genital challenge

**DOI:** 10.1038/s41541-020-00239-7

**Published:** 2020-10-01

**Authors:** Sukumar Pal, Maria I. Cruz-Fisher, Chunmei Cheng, Jennifer R. Carmichael, Delia F. Tifrea, Olga Tatarenkova, Luis M. de la Maza

**Affiliations:** Department of Pathology and Laboratory Medicine, Medical Sciences I, Room D440, University of California, Irvine, Irvine, CA 92697-4800 USA

**Keywords:** Immunology, Microbiology, Diseases

## Abstract

Implementation of a vaccine is likely the best approach to curtail *Chlamydia trachomatis* infections. The aim of this study was to determine the ability of a vaccine formulated with the recombinant major outer membrane protein (MOMP) and Th1 and Th2 adjuvants, delivered by combinations of systemic and mucosal routes, to elicit long-term protection in mice against a genital challenge with *Chlamydia muridarum*. As a negative control, mice were vaccinated with the recombinant *Neisseria gonorrhoeae* porinB, and the positive control group was immunized with *C. muridarum* live elementary bodies (EB). The four vaccines formulated with MOMP, as determined by the titers of IgG and neutralizing antibodies in serum, proliferative responses of T-cells stimulated with EB and levels of IFN-γ in the supernatants, elicited robust humoral and cellular immune responses over a 6-month period. Groups of mice were challenged genitally at 60, 120, or 180 days postimmunization. Based on the number of mice with positive vaginal cultures, number of positive cultures, length of time of shedding, and number of inclusion forming units recovered, MOMP vaccinated groups were significantly protected. To assess fertility, when the vaginal cultures became negative, female mice were caged with male mice and the outcome of the pregnancy evaluated. As determined by the number of pregnant mice and the number of embryos, two of the vaccine formulations protected mice up to 180 days postimmunization. To our knowledge this is the first subunit of Chlamydia vaccine that has elicited in mice significant long-term protection against a genital challenge.

## Introduction

*Chlamydia trachomatis* causes sexually transmitted infections worldwide and more than 130 million new cases occur annually^1,2^. This pathogenic bacterium infects epithelial cells lining the urogenital, respiratory, and gastrointestinal tracts and also the conjunctiva of the eye^3^. In women, as a result of primary acute and recurrent infections, severe long-term sequelae including pelvic inflammatory disease (PID), chronic abdominal pain, ectopic pregnancy, and infertility can occur^3–6^. Chronic infection of the eye leads to trachoma the world’s main infectious cause of blindness^7,8^. Public health programs based on diagnostic screening and antibiotic treatment have failed to control urogenital or ocular infections^9–11^. Therefore, implementation of an efficacious vaccine is a priority^12–16^. Eliciting sterilizing immunity with a vaccine is highly unlikely^17^. However, computer modeling indicates that chlamydial vaccines that are at least 50% efficacious can have a marked effect on controlling these infections^18^.

In the 1950’s, live or inactivated whole organism chlamydial vaccines were tested in humans and non-human primates to protect against trachoma^3,7^. These studies reached several conclusions. Some vaccination protocols induced protective immunity. The protection however, was found to be short-lived, usually for two to three years, and serovar/subgroup specific. Furthermore, certain vaccinated individuals developed a hypersensitivity reaction, or became more susceptible to reinfections, upon exposure to *C. trachomatis*. Although the cause of the hypersensitivity reaction is still under investigation, the likelihood that it was mediated by a component present in the whole organism resulted in the search for a *C. trachomatis* subunit vaccine^19^.

Several investigators have assessed the ability of live vaccines to elicit long-term protection against a genital challenge^20–23^. For example, Ramsey et al. vaccinated mice vaginally with live *C. muridarum* EB and challenge the animals at different times postimmunization^20^. The authors reported that mice became susceptible to reinfection, as shown by vaginal shedding, starting at 100 days following vaccination. Ramsey et al. could not evaluate the impact of the challenge on long-term sequelae since infertility resulted following vaginal vaccination^20^. Pal et al. vaccinated mice by the intranasal route using live *C. muridarum* EB and challenged the animals in the ovarian bursa at 90, 120, and 180 days postimmunization^21^. As determined by the number of mice with positive cultures and the number of *C. muridarum* inclusion forming units (IFU) recovered, immunized mice, when compared with the sham-immunized group, were protected up to 180 days. Importantly, as shown by the number of fertile mice and the number of embryos, mice were also protected against infertility. The use of a live chlamydial vaccine in humans is unlikely and therefore, there is a need to formulate an efficacious subunit vaccine.

The major outer membrane protein (MOMP) of *C. trachomatis*, is surface exposed, highly antigenic, accounts for the serotyping method used to classify this organism and therefore, likely for the serovar/subgroup protection found in the trachoma vaccine trials^24–26^. Immunization of mice with either native, or recombinant MOMP, has been shown to elicit protective responses against a genital challenge lasting for at least a month from the last immunization^27–31^. Here, to determine if a vaccine formulated with recombinant MOMP can induce long-term protection, BALB/c mice were vaccinated by several routes, using CpG-1826+Montanide ISA 720 as adjuvants. Different groups of mice were challenged in the genital tract at 60, 120, or 180 days postimmunization.

## Results

### Humoral immune responses following vaccination

**Fig. 1:**
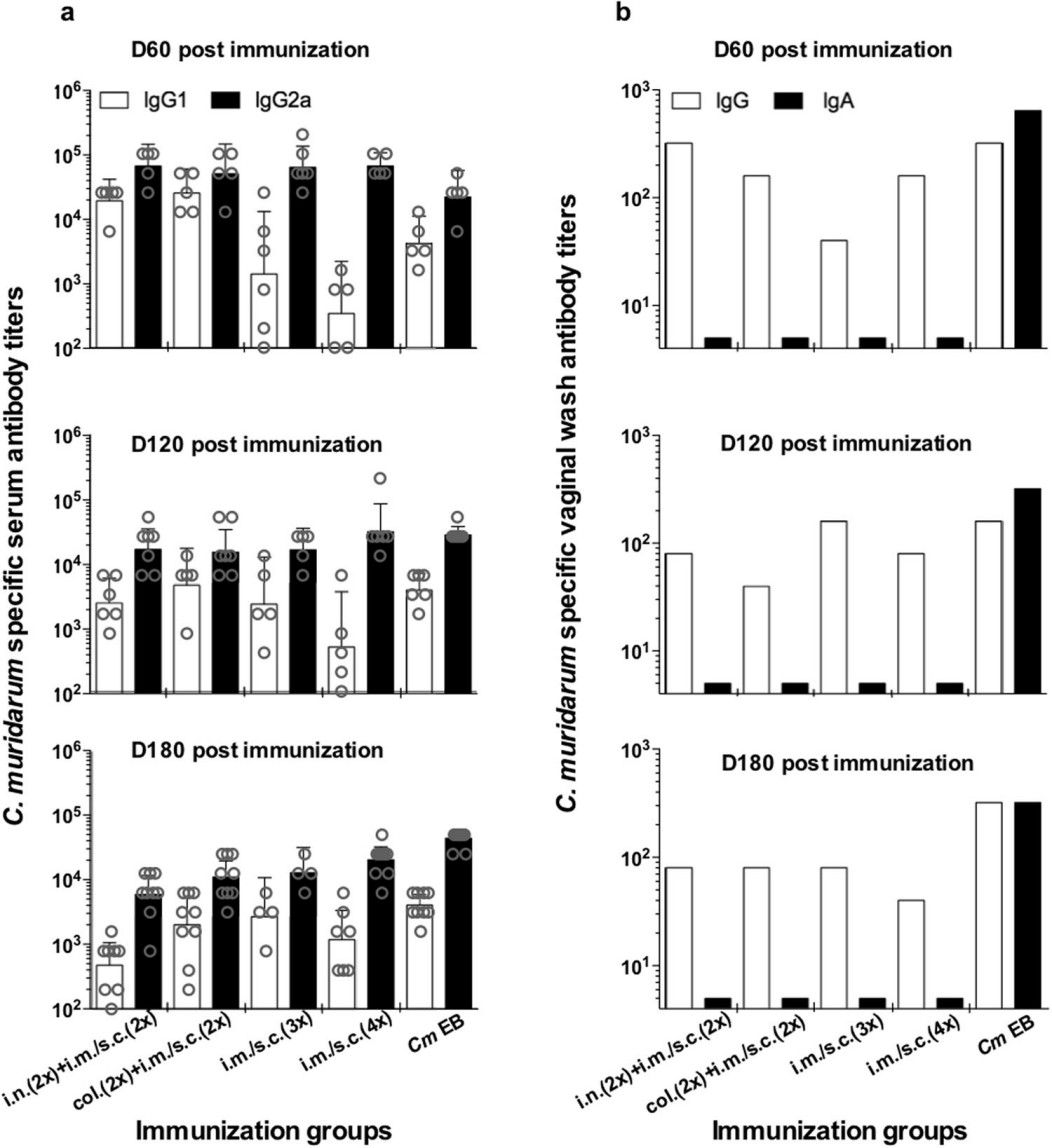
Serum and vaginal *C. muridarum-*specific antibody titers the day prior to the genital challenge. Mice were vaccinated with *C. muridarum* MOMP using different combinations of mucosal and systemic routes. CpG-1826 and Montanide ISA 720 (only systemically) were used as adjuvants. The day before the genital challenges at 60, 120 and 180 days postimmunization blood and one pool of vaginal washes were tested from each group and antibody titers determined using *C. muridarum* EB as antigen. IgG2a and IgG1 titers were determined in serum (**a**) and IgG and IgA titers in vaginal washes (**b**). Error bars represent geometric mean with 95% confidence intervals. EB elementary bodies, col colonic, i.n. intranasal, i.m. intramuscular, s.c. subcutaneous.

Mice were immunized with four different vaccine formulations and the results of the humoral immune responses to *C. muridarum* the day before each of the three vaginal challenges are shown in Fig. 1a. Overall, the highest *C. muridarum*-specific antibody titers in serum were observed at 60 days postMOMP immunization and subsequently declined overtime. For example, in mice immunized by the col. + s.c. + i.m. routes, the IgG2a geometric mean titer (GMT) at 60 days postimmunization (d.p.i.) was 51,200 and declined to 15,603 and 10,973 at 120 and 180 d.p.i., respectively. The positive control group, mice immunized once i.n. with live EB, had no significant changes in antibody titers during the complete course of the experiment.

No major differences in IgG2a antibody titers were found between the four groups of mice immunized with MOMP using different routes. For instance, the IgG2a GMT at 60 d.p.i. ranged from 67,559 to 51,200 (*P* > 0.05). In contrast, IgG1 titers were lower in mice systemically vaccinated when compared with the two groups of mice immunized by a mucosal followed by a systemic route.

The four groups of mice immunized with MOMP had higher levels of IgG2a than IgG1 at the three-time points indicative of sustained Th1-biased responses. However, since the IgG1 levels were lower in mice vaccinated only by the systemic routes, in comparison to the groups immunized by the mucosal followed by the systemic routes, the IgG2a/IgG1 ratios were different among those groups. For example, in mice immunized by the i.n. + s.c. + i.m. routes at 60 d.p.i. the IgG2a/IgG1 ratio was 3.5, while for mice vaccinated four times i.m. the ratio was 194. Live EB, used as a positive control, also elicited robust Th1 responses as determined by the IgG2a/IgG1 ratios (4 at 60 d.p.i.). The negative-control groups, mice immunized with *N. gonorrhoeae* PorB (*Ng*-PorB), did not have detectable *C*. *muridarum*-specific antibodies (<100; data not shown).

**Fig. 2:**
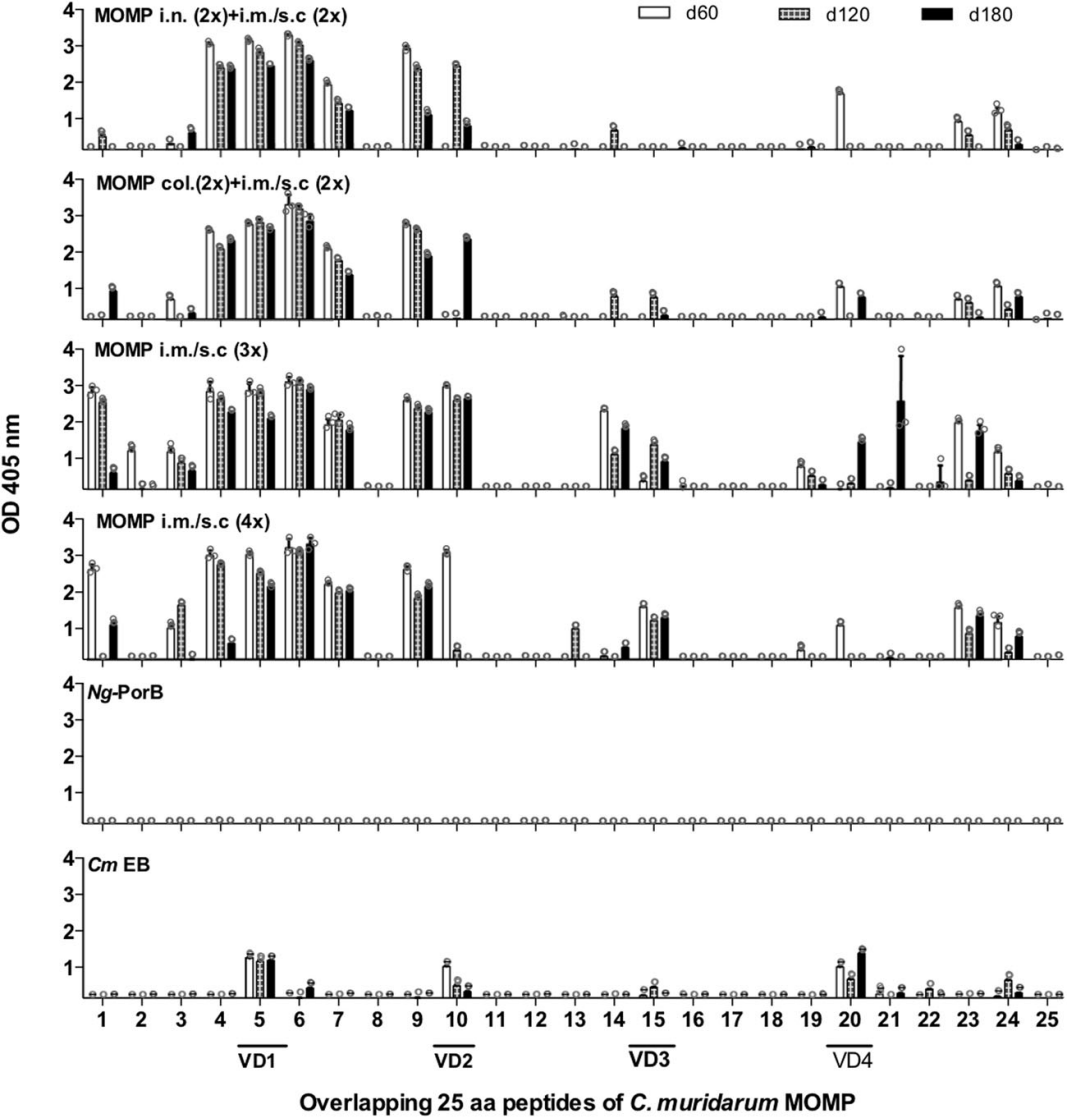
Binding of serum antibodies to synthetic *C. muridarum* MOMP peptides. Serum samples from immunized mice were collected the day before each of the genital challenges and their reactivity to 25-mer peptides corresponding to the *C. muridarum* mature MOMP were analyzed by ELISA. EB elementary bodies, col colonic, i.n. intranasal, i.m. intramuscular, s.c. subcutaneous.

Mapping of antibody responses in serum were determined using MOMP peptides. As shown in Fig. 2, all groups of mice vaccinated with MOMP elicited the most robust antibody responses to the N-terminal region of MOMP including CD1, VD1, CD2, and VD2. Weaker responses occurred to VD3, VD4, and CD5. Antibody responses to VD3 and CD5 were higher in mice vaccinated by systemic routes than in those immunized with a combination of mucosal plus systemic routes. Mice immunized with live EB-mounted antibody responses to the four VD and CD5.

**Fig. 3:**
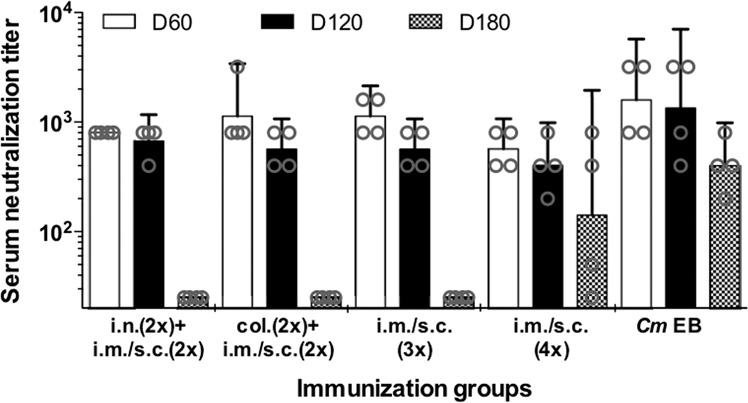
Serum *C. muridarum*-specific neutralization titers the day prior to the genital challenges. Mice were vaccinated with *C. muridarum* MOMP, using different combinations of mucosal and systemic routes. CpG-1826 and Montanide ISA 720 (only systemically) were used as adjuvants. The day before the genital challenges at 60, 120, and 180 days postimmunization blood was collected and neutralizing antibody titers determined using *C. muridarum* EB as antigen. Error bars represent geometric mean titers with 95% confidence intervals. EB elementary bodies, col colonic, i.n. intranasal, i.m. intramuscular, s.c. subcutaneous.

In vitro neutralization titers were measured in serum samples collected before each of the three challenges. As shown in Fig. 3, the GMT were initially similar for the four groups of animals vaccinated with MOMP. Titers declined from day 60 to day 180 postimmunization for the four groups. At 180 days only mice immunized four times i.m. + s.c. had a positive neutralization titer. The positive control immunized with live EB that had the highest neutralizing titers that also declined over the 180 days. The four negative controls that received *Ng*-PorB were all negative (<50; data not shown).

The GMT of *C. muridarum*-specific IgG antibodies in vaginal washes of mice immunized with MOMP were low (Fig. 1b). The highest IgG GMT (320) was observed in the i.n. (2×) + i.m./s.c. (2×) immunized group at 60 d.p.i. The other three groups had lower titers (160 and 40 at that time point). No IgA was detected in vaginal washes in any of the four groups vaccinated with MOMP. The live EB-immunized positive-control group had the highest IgA and IgG antibody levels in the vaginal washes at the three challenge days. The *Ng*-PorB-immunized negative-control groups had no detectable antibody titers in the vagina (<10; data not shown).

### Cell-mediated immune responses elicited by vaccination

**Fig. 4:**
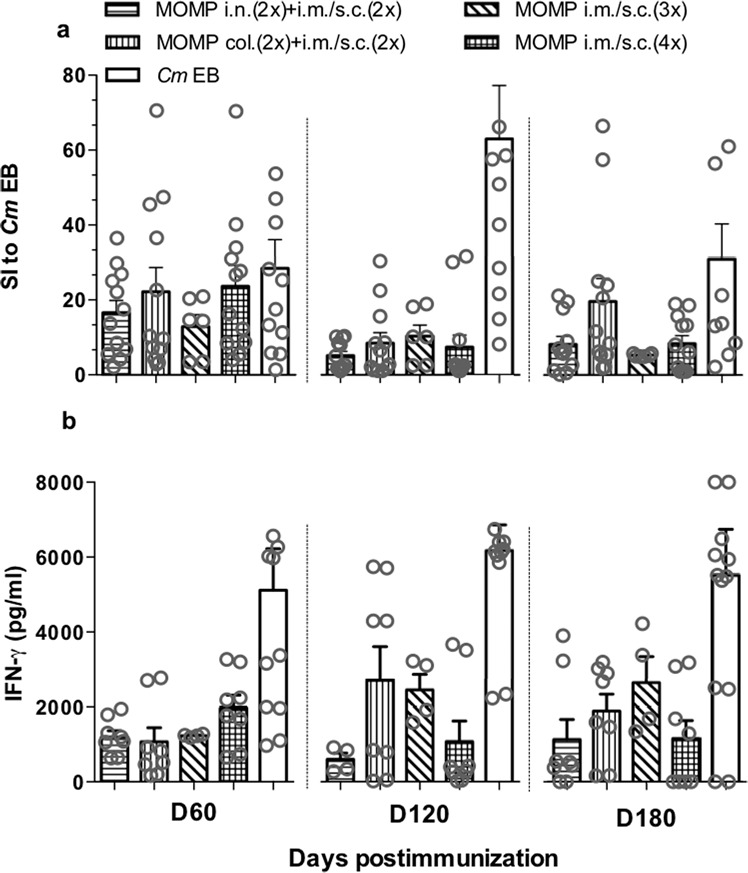
Cell-mediated immune responses of vaccinated mice the day before the genital challenges at 60, 120 and 180 days following immunization. Spleen T-cells, collected the day before the genital challenges, were stimulated with *C. muridarum* EB and the proliferative responses (**a**) and levels of IFN-γ (**b**) secretion were determined. As a negative background control, T-cells were stimulated with minimal essential medium. Error bars represent mean with one standard error. EB elementary bodies, col colonic, i.n. intranasal, i.m. intramuscular, s.c. subcutaneous, SI stimulation index.

The cellular proliferative responses were determined using spleen T-cells stimulated with *C. muridarum* EB. As shown in Fig. 4a, the four groups of mice immunized with MOMP had significant proliferative immune responses at 60, 120, and 180 d.p.i. when compared with animals vaccinated with *Ng*-PorB. The proliferative responses were similar among the four groups of mice at the three time points tested. For instance, at 60 d.p.i. the Δcpm and the stimulation index (SI) for each group of mice were: i.n. + i.m. + s.c. 2385 (SI: 16.6); col. + i.m. + s.c. 2042 (SI: 22.2); 3× i.m.+ s.c. 2229 (SI: 12.9) and 4× i.m.+ s.c. 2615 (SI 23.7). At 180 d.p.i. the values were 2028 (SI: 8.2), 2955 (SI: 19.6), 7851 (SI: 5.3), and 2631 (SI: 8.4) respectively, indicative that there were sustained cellular immune responses.

Levels of IFN-γ were measured in supernatants from EB-stimulated T-cells (Fig. 4b). There were no major changes overtime in levels of IFN-γ in any of the four groups vaccinated with MOMP. Positive control mice immunized with live *C. muridarum* EB had the most robust proliferative responses and levels of IFN-γ. Mice immunized with *Ng*-PorB had low proliferative responses and produced IFN-γ below the level of detection (<15 pg/ml) (data not shown).

### Vaginal cultures

**Fig. 5:**
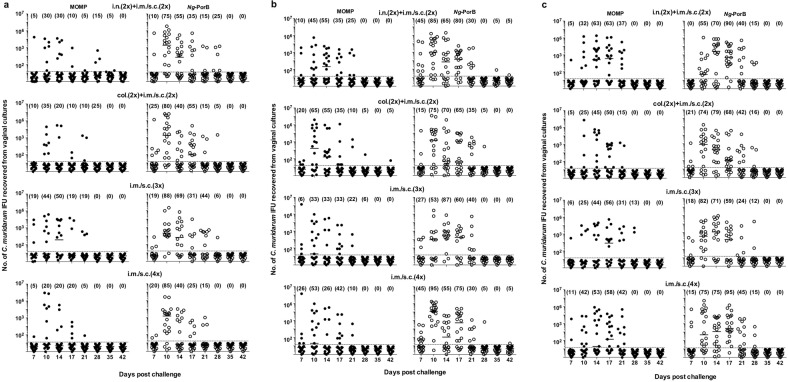
Vaginal cultures of mice following intrabursal challenges with *C. muridarum* at 60, 120, and 180 days postimmunization. Mice were immunized with recombinant *C. muridarum* MOMP, or with *N. gonorrhoeae* porB as a negative control antigen, using different combinations of mucosal and systemic routes. CpG-1826 and Montanide ISA 720 (only systemically) were used as adjuvants. At 60 (**a**), 120 (**b**) and 180 (**c**) days postimmunization, mice were challenged in the left ovarian bursa with *C. muridarum* and vaginal cultures were collected for a period of 6 weeks. EB elementary bodies, col colonic, i.n. intranasal, i.m. intramuscular, s.c. subcutaneous. Each dot represents a mouse. Horizontal bars represent median IFU numbers. Numbers in brackets correspond to percentage of mice with positive vaginal cultures. Limit of detection (<2 IFU/culture).

**Fig. 6:**
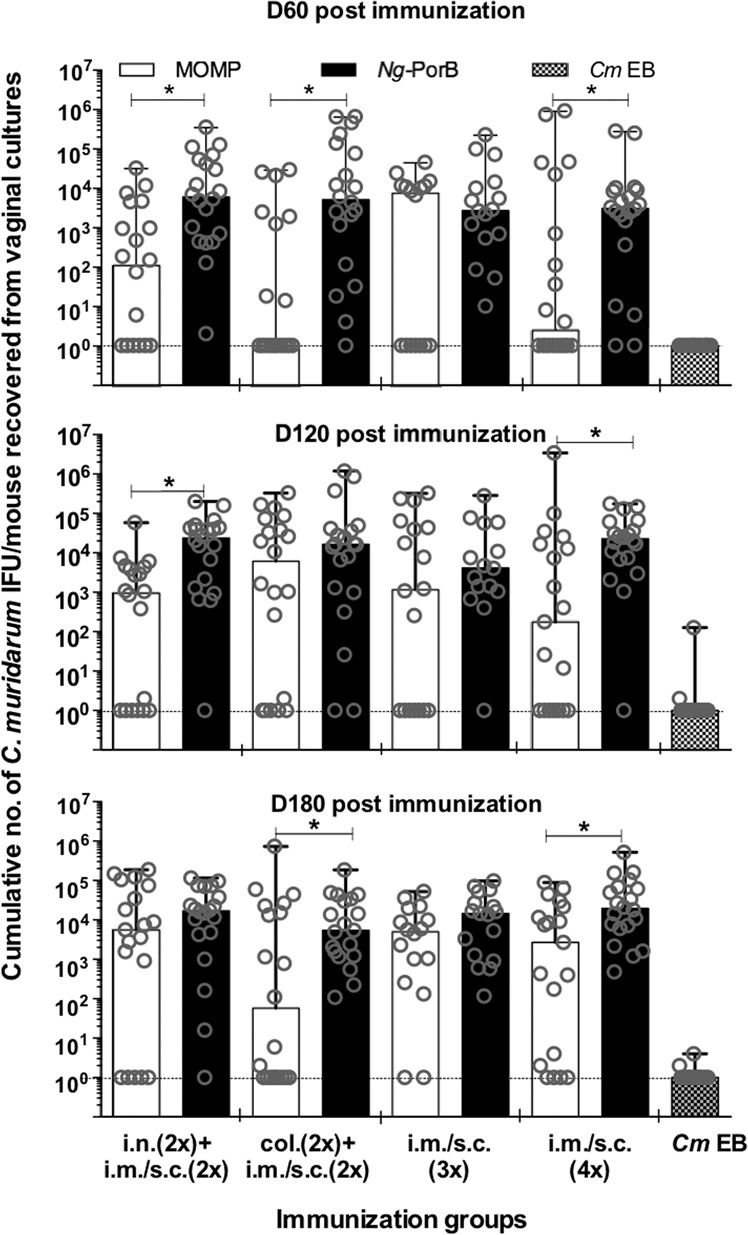
Cumulative number of *C. muridarum* IFU collected per mouse from vaginal cultures at 60, 120, and 180 days postimmunization. The number of *C. muridarum* IFU collected from vaginal cultures at 60, 120, and 180 days postchallenge were totaled. Bars represent the median number of *C. muridarum* IFU/mouse and error bars represent range. Symbol * indicates *P* value is less than 0.05 by the Mann–Whitney *U*-test. EB elementary bodies, col colonic, i.n. intranasal, i.m. intramuscular, s.c. subcutaneous.

At 60, 120, or 180 days after the last immunization, mice were challenged in the left ovarian bursa with 10^5^ IFU of *C. muridarum* and the course of the infection was followed with vaginal cultures for a 6-week period (Table 1 and Figs. 5, 6). Four parameters were used to ascertain protection against the challenge: percentage of mice with positive vaginal cultures, total number of positive vaginal cultures, median length of time (in days) of vaginal shedding, and number of *C. muridarum* IFU recovered. Overall, as determined by these parameters, the four groups of mice immunized with MOMP were significantly protected at the three time points, when compared with their respective *Ng*-PorB immunized groups.

**Table 1:** Vaginal shedding of mice genitally challenged at 60, 120, or 180 days postimmunization.

Antigen	Immunization route	# + mice shed/total (%+), # + cultures/total (%+), and median # days (range) to clear infection.								
		D60 postimmunization			D120 postimmunization			D180 postimmunization		
		# + mice/total (%+)	# + cultures/total (%+)	Median # days to -culture	# + mice/total (%+)	# + cultures/total (%+)	Median # days to -culture	# + mice/total (%+)	# + cultures/total (%+)	Median # days to -culture^e^
MOMP	i.n. (2×) + i.m./s.c. (2×)	12/20 (60)^a^	20/160 (13)^a^	16 (7–35)^c^	13/20 (65)^a^	34/160 (21)^a^	17 (7–28)^c^	14/19 (74)	38/152 (25)	21 (7–28)
*Ng*-PorB	i.n. (2×) + i.m./s.c. (2×)	20/20 (100)	43/160 (27)	17 (14–35)	19/20 (95)	63/160 (39)	21 (7–42)	19/20 (95)	54/160 (34)	21 (7–35)
MOMP	col. (2×) + i.m./s.c. (2×)	8/20 (40)^a^	15/160 (9)^a^	7 (7–28)^c^	16/20 (80)	39/160 (24)	17 (7–42)^d^	12/20 (60)^a^	28/160 (18)^a^	19 (7–28)^d^
*Ng*-PorB	col. (2×) + i.m./s.c. (2×)	19/20 (95)	43/160 (27)	21 (7–35)	18/20 (90)	53/160 (33)	21 (7–35)	19/19 (100)	57/152 (38)	21 (17–42)
MOMP	i.m./s.c. (3×)	9/16 (56)^a^	24/152 (16)^a^	16 (7–28)^c^	11/18 (61)^a^	24/144 (17)^a^	19 (7–35)	14/16 (88)^b^	28/128 (22)^d^	21 (7–35)
*Ng*-PorB	i.m./s.c. (3×)	16/16 (100)	41/152 (27)	21 (14–35)	14/15 (93)	50/120 (42)	21 (7–28)	17/17 (100)	45/136 (33)	21 (14–35)
MOMP	i.m./s.c. (4×)	10/20 (50)^a^	17/160 (11)^a^	11 (7–28)^c^	12/19 (63)^a^	30/152 (20)^a^	14 (7–28)^c^	15/19 (79)^a^	39/152 (26)^a^	21 (7–28)^d^
*Ng*-PorB	i.m./s.c. (4×)	18/20 (90)	37/160 (23)	16 (7–28)	19/20 (95)	52/160 (33)	21 (7–42)	20/20 (100)	64/160 (40)	28 (21–35)
*Cm* EB	i.n.	0/20 (0)	0/160 (0)	7 (7–7)	2/20 (10)	2/160 (1)	7 (7–17)	2/20 (10)	2/160 (2)	7 (7–10)

^a^*P* < 0.05 by Fisher’s exact test compared with the corresponding *Ng*-PorB immunized mice.

^b^*P* < 0.1 by Fisher’s exact test compared with the corresponding *Ng*-PorB immunized mice.

^c^*P* < 0.05 by the Mann–Whitney *U*-test compared with the corresponding *Ng*-PorB immunized mice.

^d^*P* < 0.1 by the Mann–Whitney *U*-test compared with the corresponding *Ng*-PorB immunized mice.

^e^The first culture was collected at 7 days post-challenge.

There was a consistent decrease in the percentage of mice with positive vaginal cultures in groups vaccinated with MOMP versus controls immunized with *Ng*-PorB (Table 1 and Fig. 5). For the three challenge days, 40–88% of mice vaccinated with MOMP had positive vaginal cultures compared to 90–100% of control animals immunized with *Ng*-PorB. The four groups vaccinated with MOMP and challenged at 60 d.p.i., had a significantly lower percentage of mice with positive cultures when compared with their respective *Ng*-PorB immunized animals (*P* < 0.05). As determined by percentage of mice with positive vaginal cultures, except for the group immunized col + i.m. + s.c., there was a progressive decrease in protection when comparing mice challenged at 60, 120, and 180 d.p.i. Approximately 50% of the mice vaccinated with MOMP and challenged at 60 d.p.i. had positive vaginal cultures while ~75% of those challenged at 180 d.p.i. had positive cultures. Negative control mice immunized with *Ng*-PorB had consistently higher positivity rates (~96%). Positive control mice, receiving EB i.n., had very solid protection.

The total number of positive vaginal cultures was lower in mice vaccinated with MOMP in comparison to negative control animals (Table 1 and Fig. 5a–c). In general, mice vaccinated with MOMP had half the number of positive vaginal cultures that those immunized with *Ng*-PorB. For instance, of the mice immunized with MOMP using the col. + i.m. + s.c. routes, at 60 d.p.i., 9% (15/160) had positive vaginal cultures while the controls immunized with *Ng*-PorB had 27% (43/160) positive vaginal cultures (*P* < 0.05). The protection slowly declined as the time to challenge increased. For the same group at 180 d.p.i., the numbers were 18% (28/160) and 38% (57/152), respectively (*P* < 0.05).

The shortest median length of time (days) of shedding was observed in mice vaccinated four times i.m./s.c., or by the col. + i.m./s.c. routes (Table 1 and Fig. 5). In mice vaccinated four times i.m./s.c with MOMP significant differences were observed between groups challenged at 60 (median 11; range 7–28) and 120 (median 14; range 7–28) d.p.i. when compared with animals immunized with *Ng*-PorB (median 16; range 7–28) and (median 21; range 7–42), respectively (*P* < 0.05). For these two groups vaccinated with MOMP at 180 d.p.i. the differences were approaching significance (*P* < 0.1) when compared to mice immunized with *Ng*-PorB.

As determined by the number of *C. muridarum* IFU recovered from the vaginal swabs, except for the group immunized three times i.m. + s.c., mice vaccinated with MOMP were protected (Fig. 6). Mice vaccinated four times im. + s.c. were better protected when compared to the other MOMP immunized groups. For the group immunized four times i.m. + s.c. the median number of *C. muridarum* IFU/mouse over the 6 weeks of culture were, <2 (<2–889,082), 200 (<2-3,443,500) and 2700 (<2–83,420) at 60, 120, and 180 d.p.i., respectively. All these values were significantly lower than 3000 (<2–277,008), 22,600 (<2–175,570) and 19,000 (50–523,800) for the group immunized with *Ng*-PorB (*P* < 0.05).

### Fertility results

To ascertain the ability of MOMP to protect against long-term sequelae, specifically infertility, mice were mated seven weeks after the intrabursal challenge. As shown in Table 2, all but the groups vaccinated via the i.n. + i.m. + s.c. routes with MOMP, and challenged at 60 and 180 d.p.i., showed higher bilateral fertility rates than those immunized with *Ng*-PorB, although differences for most groups were not statistically significant. For example, for mice immunized with MOMP by the col. + i.m. + s.c. routes the fertility rates were 60, 40 and 58%, while for the negative controls were 30, 20, and 5%, at 60, 120, and 180 d.p.i., respectively. Similarly, the fertility rates for mice vaccinated with MOMP four times i.m. + s.c. and challenged at 60, 120, and 180 d.p.i. were 40, 58, and 37%, while for animals immunized with *Ng*-PorB were 25, 16, and 10%, respectively. Bilateral fertility in mice immunized with EB was similar to the fertility control group (85–95%).

**Table 2:** Fertility results of mice genitally challenged at 60, 120, or 180 days post-immunization.

Antigen	Immunization route	# bilateral fertile mice/total (%+)			Mean # embryos (±1 SD) in left uterine horn^a^			Mean # embryos (±1 SD) in right uterine horn		
		D60	D120	D180	D60	D120	D180	D60	D120	D180
MOMP	i.n. (2×) + i.m./s.c. (2×)	2/20 (10)	9/20 (45)^b^	6/18 (33)	0.3 ± 0.8	1.1 ± 1.5^d^	1.3 ± 1.6	2.9 ± 2.8	2.7 ± 1.8	2.7 ± 2.1
*Ng*-PorB	i.n. (2×) i.m./s.c. (2×)	4/20 (20)	1/20 (5)	7/20 (35)	1.2 ± 1.6	0.3 ± 0.8	1.2 ± 2.0	1.9 ± 2.1	1.7 ± 2.3	3.0 ± 2.1
MOMP	col. (2×) + i.m./s.c. (2×)	12/20 (60)	8/20 (40)	11/19 (58)^b^	1.4 ± 1.3	1.1 ± 1.5	1.6 ± 1.8^d^	3.4 ± 1.8	3.6 ± 2.0	2.8 ± 1.7^d^
*Ng*-PorB	col. (2×) + i.m./s.c. (2×)	6/20 (30)	4/20 (20)	1/19 (5)	1.3 ± 2.2	0.8 ± 1.5	0.1 ± 0.2	3.0 ± 2.5	3.0 ± 2.2	1.5 ± 1.9
MOMP	i.m./s.c. (3×)	8/16 (50)^b^	7/18 (39)	2/16 (13)	1.1 ± 1.2^c^	1.0 ± 1.4	0.6 ± 1.2	2.6 ± 2.0	3.1 ± 1.7^e^	1.8 ± 1.7
*Ng*-PorB	i.m./s.c. (3×)	0/16 (0)	4/15 (27)	1/17 (6)	0 ± 0	0.7 ± 1.3	0.5 ± 1.1	2.5 ± 2.5	1.9 ± 1.8	1.9 ± 1.9
MOMP	i.m./s.c. (4×)	8/20 (40)	11/19 (58)^b^	7/19 (37)^c^	2.1 ± 2.0^e^	1.3 ± 1.5	1.3 ± 1.8^d^	2.9 ± 2.2	3.0 ± 1.3^d^	1.9 ± 1.8
*Ng*-PorB	i.m./s.c. (4×)	5/20 (25)	3/19 (16)	2/20 (10)	0.9 ± 1.8	1.2 ± 2.0	0.1 ± 0.2	2.7 ± 2.2	1.2 ± 1.8	2.2 ± 2.2
*Cm* EB	i.n.	18/20 (90)	17/20 (85)	17/20 (85)	2.6 ± 1.6	2.0 ± 1.4	2.2 ± 1.4	3.4 ± 2.1	2.7 ± 1.8	2.3 ± 1.6
Fertility control		18/20 (90)	18/19 (95)	18/20 (90)	3.4 ± 1.8	3.5 ± 2.2	2.2 ± 1.9	3.4 ± 2.4	3.9 ± 1.1	2.2 ± 1.5

^a^Left ovarian bursae were challenged with 10^5^ IFU of *C. muridarum*.

^b^Significant (*P* < 0.05) by the Fisher’s Exact test compared to the corresponding *Ng*-PorB immunized mice.

^c^Significant (*P* < 0.1) by the Fisher’s Exact test compared to the corresponding *Ng*-PorB immunized mice.

^d^Significant (*P* < 0.05) by the Student’s *t*-test compared to the corresponding *Ng*-PorB immunized mice.

^e^Significant (*P* < 0.1) by the Student’s *t*-test compared to the corresponding *Ng*-PorB immunized mice.

A similar trend was observed when analyzing the number of embryos in the left uterine horn, the challenged site. The mean number of embryos in the left uterine horn for mice vaccinated four times with MOMP i.m. + s.c. and challenged at 60, 120, and 180 d.p.i. were 2.1, 1.3, and 1.3, respectively, while for animals immunized with *Ng*-PorB were 0.9, 1.2, and 0.1, respectively. The mean number of embryos in the right uterine horn was also consistently higher in mice immunized with MOMP versus *Ng*-PorB. Controls immunized i.n. with EB had similar number of embryos as the fertility control.

### Cumulative protective efficacy

Humans are exposed to pathogens at different times following immunization and therefore, vaccine efficacy is calculated based on cumulative data independently of the time lapse between immunization and exposure. Thus, to determine the efficacy of the MOMP vaccine, we analyzed the cumulative results for the three challenge dates (Table 3).

**Table 3:** Overall protective efficacy of four vaccination protocols following three genital challenges at 60, 120, or 180 days post-immunization.

Antigen	Immunization route	# + mice/total (%+)	# + vaginal cultures/total (%+)	Median # days to -culture	Cumulative median # IFU shed/mouse (range) ×10^3^	# bilateral fertile mice (%)	Mean # embryos (1 ± SD) in uterine horns		
							Left	Right	Both
MOMP	i.n. (2×) + i.m./s.c. (2×)	39/59 (66)^a^	92/472 (19)^a^	17 (7–35)^b^	0.9 (<0.002–190)^b^	17/58 (29)	0.9 ± 1.4	2.7 ± 2.0	3.6 ± 2.4
*Ng*-PorB	i.n. (2×) + i.m./s.c. (2×)	58/60 (97)	160/480 (33)	21 (7–42)	15.5 (<0.002–348)	12/60 (20)	0.9 ± 1.6	2.2 ± 2.2	3.1 ± 2.8
MOMP	col (2×) + i.m./s.c. (2×)	36/60 (60)^a^	82/480 (17)^a^	16 (7–42)^b^	0.09 (<0.002–738)^b^	31/59 (53)^a,g^	1.3 ± 1.5^c^	3.2 ± 1.8^d^	4.6 ± 2.4^c,e,f^
*Ng*-PorB	col (2×) + i.m./s.c. (2×)	56/59 (95)	153/472 (32)	21 (7–42)	8.0 (<0.002–1202)	11/59 (19)	0.7 ± 1.6	2.5 ± 2.3	3.3 ± 2.9
MOMP	i.m./s.c. (3×)	34/50 (68)^a^	76/424 (18)^a^	17 (7–35)^b^	5.0 (<0.002–327)	17/50 (34)^a^	0.9 ± 1.2^c^	2.5 ± 1.8	3.4 ± 2.3^d^
*Ng*-PorB	i.m./s.c. (3×)	47/48 (98)	136/408 (33)	21 (7–35)	4.7 (<0.002–283)	5/48 (10)	0.4 ± 1.0	2.1 ± 2.0	2.5 ± 2.2
MOMP	i.m./s.c. (4×)	37/58 (64)^a^	86/464 (19)^a^	17 (7–28)^b^	0.14 (<0.002–3449)^b^	26/58 (45)^a^	1.6 ± 1.8^c,e,f^	2.6 ± 1.8	4.1 ± 2.6^c^
*Ng*-PorB	i.m./s.c. (4×)	57/60 (95)	153/480 (32)	21 (7–42)	10.3 (<0.002–524)	10/59 (17)	0.7 ± 1.6	2.0 ± 2.2	2.7 ± 2.8
*Cm* EB	i.n.	4/60 (7)	4/480 (0.8)	7 (7–17)	<0.002 (<0.002–0.1)	52/60 (87)	2.2 ± 1.5	2.8 ± 1.9	5.0 ± 2.7
Fertility control	–	NT	NT	NT	NT	54/59 (92)	3.0 ± 2.1	3.2 ± 1.9	6.2 ± 3.3

*NT* not tested.

^a^*P* < 0.05 by the Fisher’s Exact test compared to the corresponding *Ng*-PorB immunized group.

^b^*P* < 0.05 by the Mann–Whitney *U*-test compared to the corresponding *Ng*-PorB immunized group.

^c^*P* < 0.05 by the Student’s *t*-test compared to the corresponding *Ng*-PorB immunized group.

^d^*P* < 0.1 by the Student’s *t*-test compared to the corresponding *Ng*-PorB immunized group.

^e^*P* < 0.05 by the Student’s *t*-test compared to the MOMP i.m./s.c. (3×) immunized group.

^f^*P* < 0.05 by the Student’s *t*-test compared to the MOMP i.n. (2×) + i.m./s.c. (2×) immunized group.

^g^*P* < 0.05 by the Fisher’s exact test compared to the MOMP i.n. (2×) + i.m./s.c. (2×) immunized group.

Based on the number of mice that had positive vaginal cultures, all groups of mice vaccinated with MOMP were significantly protected in comparison to the controls immunized with *Ng*-PorB. Only 60–68% (36/60–34/50) of mice vaccinated with MOMP had positive vaginal cultures a decrease of ~35% in positivity rate, when compared to 95–98% (57/60–47/48) of the animals immunized with *Ng*-PorB (*P* < 0.05). Only 7% (4/66) of the control EB-immunized mice had positive cultures.

The number of positive vaginal cultures for MOMP-vaccinated mice was also significantly lower when compared with their respective *Ng*-PorB immunized group (Table 3). For example, in mice vaccinated with MOMP the number of positive cultures ranged from 17% (82/480) to 19% (92/472), while for mice immunized with *Ng*-PorB it ranged from 32% (153/472) to 33% (160/480) (*P* < 0.05). In the EB-immunized group 0.8% (4/480) of the cultures were positive.

The cumulative median time (days) to negative culture was calculated for all groups (Table 3). The four groups of mice vaccinated with MOMP had significantly shorter median time of vaginal shedding. The median number of days was 16 or 17 days for mice vaccinated with MOMP while it was 21 days for animals immunized with *Ng*-PorB (*P* < 0.05). Control animal immunized with EB had a median number of days to clearance of 7 (first day of culture after the challenge).

Based on the number of IFU shed per mouse, animals vaccinated with MOMP were also protected compared with the negative controls. The media number of IFU shed/mouse ranged from 90 to 5000 for mice vaccinated with MOMP versus 4700 to 15,500 for the negative controls immunized with *Ng*-PorB (Table 3) (*P* < 0.05). The only MOMP vaccinated group that was not significantly different from the *Ng*-PorB control was the one immunized three times i.m. + s.c. Most mice immunized with EB had a number of IFU below the limit of detection.

Significant protection against bilateral infertility and number of embryos in the left uterine horn, were obtained in three of the four groups vaccinated with MOMP when compared with their respective controls immunized with *Ng*-PorB (Table 3). For example, for the group vaccinated by the col.+ i.m. + s.c. routes, bilateral fertility was observed in 53% (31/59) of the MOMP-vaccinated mice while in the *Ng*-PorB group only 19% (11/59) of the animals were fertile (*P* < 0.05). Also, for mice vaccinated four times i.m. + s.c. with MOMP the percentage of animals with bilateral fertility was 45% (26/58) and in the control group immunized with *Ng*-PorB it was 17% (10/59) (*P* < 0.05). Controls immunized i.n. with EB had similar number of bilaterally fertile mice (87%) when compared with the fertility controls (92%) (*P* > 0.05).

Except for mice vaccinated with MOMP by the i.n. + i.m. + s.c. routes, the other three groups were protected against infertility at the challenge site, the left uterine horn (Table 3). In animals vaccinated with MOMP by the col. + i.m. + s.c. routes the mean number of embryos in the left uterine horn was 1.3 versus 0.7 for mice immunized *Ng*-PorB (*P* < 0.05). In mice vaccinated four times i.m. + s.c. with MOMP, the mean number of embryos in the left uterine horn was 1.6 compared with 0.7 in mice immunized with *Ng*-PorB (*P* < 0.05). It is interesting to note that although not statistically significantly different, the four groups of mice vaccinated with MOMP also had higher number of mice in the right uterine horn than animals immunized with *Ng*-PorB. The total number of embryos in mice vaccinated by the col. + i.m. + s.c. (4.6) and i.m./s.c. (4×) (4.1) routes, was not significantly different from the number of embryos in the positive EB-immunized control (5.0) (*P* > 0.05).

## Discussion

Here we sought to determine if a subunit vaccine can elicit long-term protection in female mice against a *C. muridarum* genital challenge. We tested four protocols using recombinant MOMP as the antigen, CpG-1826, and Montanide ISA 720 as the adjuvants and several combinations of mucosal and systemic routes for immunization. All four formulations elicited significant cellular and humoral immune responses. The immune responses were robust at 60 days postimmunization and then, in particular the humoral responses, declined by 180 days postimmunization. As determined by vaginal shedding and infertility, the most effective vaccine formulations were those delivered four times by a combination of colonic plus intramuscular and subcutaneous routes, or by systemic routes only. The fertility period for human females expands approximately from 15 to 45 years of age and therefore, a chlamydial vaccine does not necessarily has to provide lifetime protection against infertility^21^. In this model, as determined by the number of pregnant animals and number of embryos, mice were protected for a period of 180 days, equivalent to approximately a third of the life span of mice, suggesting that it should be possible to formulate vaccines that protect females during their fertile years. To our knowledge, this is the first time that a subunit vaccine has been shown to induce significant long-term protection against a chlamydial genital challenge.

*C. trachomatis* affects mainly individuals in their early years of sexual activity^2,32^. Most females are infected with *C. trachomatis* during their teens and early twenties and some of them develop long-term sequelae following an acute primary or a reinfection^5,6,33,34^. In older individuals, as a result of acquired immunity, changes in sexual behavior and/or physiological maturation, the rates of sexually transmitted infections, including *Chlamydia*, decline^2–4^. Mice, like humans, also become more resistant to a *C. muridarum* infection as they age, indicative of physiological maturation^35^. In a study by Westrom et al.^34^, of 141 women that developed tubal infertility, 79 became infertility after a single episode of PID, 36 after two events and 26 after three or more episodes of PID. Thus, like in the case of human papillomavirus (HPV) infections, in order to protect a person when they are at the highest risk, a vaccine needs to be delivered before the individual reaches sexual maturity and they suffer their first PID episode^36,37^. Here, we started immunization at 3 weeks of age with the goal of achieving protection by the time mice reach sexual maturity at 7–9 weeks of age.

The intrabursal model used for these studies has limitations since it does not parallel the natural route of infection and the mechanisms of protection in the lower and upper genital tract are different^38^. However, a direct challenge at the site where protection is more critically needed, may provide a more stringent proof of vaccine efficacy^39^. Also, challenging mice in one ovarian bursa, allows to determine the ability of *Chlamydia* to disseminate to the other ovarian bursa and helps evaluate the efficacy of the vaccine by comparing the number of embryos in both uterine horns^39^.

Unlike in the intrabursal model, to increase susceptibility to infection in the intravaginal and the transcervical models, mice are treated with medroxyprogesterone before they are challenged^40,41^. Progesterone has a significant immunomodulatory activity in humans and mice. This sex hormone induces a shift from Th1 to Th2 responses^42^. In case of *C. muridarum* infections, a Th1 response is considered to be necessary for protection^12,43^. Therefore, treating mice with progesterone, before they are intravaginally or transcervically challenge, can abrogate the protective effect of a vaccine by altering the immune responses^41,44,45^. This limitation has been shown in a animals model of chlamydia and herpes simplex virus infection. Treatment with progesterone blocked the protective responses following immunization^46–48^. Similarly, treatment of nonhuman primates with progesterone before an intravaginal challenge with the simian immunodeficiency virus negated the protection elicited by the vaccine^49^. Thus, in our opinion, in spite of some drawbacks, the use of the intrabursal model offers some advantages over the intravaginal and transcervical mouse models^40,50,51^.

Subunit vaccines have very limited, if any, intrinsic adjuvanticity and therefore, there is a need to formulate the antigen with adjuvants. Using several experimental models it has been determined that protection of mice against a secondary genital challenge with *C. muridarum* requires CD4 Th1 cells and neutralizing antibodies while the role of CD8 cells is still under investigation^12,43^. Thus, here we used a combination of CpG-1826, a Th1 adjuvant, with Montanide ISA 720, a Th2 adjuvant, to elicit both cellular and humoral immune responses. In this experiment, humoral immune responses declined over time in all groups except the positive controls inoculated with live EB. Interestingly, the decline in levels of IgG2a was fairly consistent in the four groups immunized with MOMP. In contrast, IgG1 titers declined in mice vaccinated by a combination of mucosal and systemic routes while they remained fairly constant in mice immunized only systemically. In the systemically immunized groups however, the initial levels of IgG1 were low compared with those from mice immunized by combined routes. Mapping of the humoral responses using synthetic peptides to MOMP, shows that most of the antibodies bind to the VD. Antibody levels to the VD are low in the EB-immunized group versus animals vaccinated with MOMP. Protection however is more robust in the EB-immunized group suggesting that during a natural infection some anti-MOMP antibodies are conformation dependent and thus, not detected by this assay. Neutralizing antibodies in serum also declined overtime in all groups probably accounting for part of the loss of protection. Only mice immunized four times systemically, and the positive control, had neutralizing antibodies at D180 postimmunization. In the vaginal washes IgG titers also declined overtime although the decline was not marked. Only controls immunized with EB had IgA antibodies in the vaginal washes. The role of IgA in protection is still under investigation. Although both in humans and mice there are reports suggesting a protective role of IgA in the vaginal mucosal, Morrison and Morrison^52^ found that resolution of a primary and a secondary vaginal *C. muridarum* infection was not different in IgA−/− versus IgA+/+ mice^52–54^. The relative stability of antibody titers in serum and vaginal washes in the EB control group, likely reflects the establishment of a *C. muridarum* chronic colonization of the gastrointestinal tract and other organs^55^.

Cellular immune responses, as determined by T-cell stimulation and levels of IFN-γ in the supernatants from EB-stimulated T-cells, were fairly constant for all the groups over the 180 days of evaluation. As expected, positive control mice immunized i.n. with live EB had the most robust and consistent cell-mediated immune responses. Therefore, the decline in protection overtime better correlated with titers of neutralizing antibodies than with levels of cell-mediated immune responses.

The route of administration of a vaccine influences the strength and nature of immune responses^56,57^. The i.m. route is the one most commonly used to vaccinate humans against pathogenic organisms independent of their site of entry. For example, the HPV, influenza and pneumococcal vaccines are delivered i.m. to protect against mucosal pathogens^36,58,59^. The same route is also used to vaccinate against *Clostridium tetani* and hepatitis B virus, both systemic pathogens^60,61^. The portal of entry of *Chlamydia* can involve several sites including the conjunctiva and the urogenital, respiratory and gastrointestinal tracts^4,62^. Therefore, identification of routes of immunization that can elicit strong immune responses at various mucosal sites is critical if we want to formulate an efficacious vaccine against this pathogen. The immunization route is particularly important because due to the compartmentalization of the mucosal immune system, stimulation of the various mucosal inductive sites results in an uneven distribution of immune responses at the various effector sites^57,63,64^. Overall, the most effective way to induce an immune response at a specific effector site is to locally administer the immunization, or perhaps, stimulate sites with related lymph drainage^57,65,66^.

Among mucosal routes, intranasal immunization with live and subunit chlamydial vaccines, has been shown to induce protection against chlamydial respiratory and genital challenges in mice^21,39,67,68^. Unfortunately, the use of the i.n. route for immunization can result in significant negative secondary effects. For example, cases of facial (Bell’s) paralysis were reported following the i.n. delivery of the influenza vaccine^69,70^. The immediate proximity of the olfactory epithelium, located in the middle of the roof of the nasal cavity, to the central nervous system is a significant concern for delivering vaccines by the i.n. route. For these reasons, here, in addition to the i.n., we also explored the colonic route, a recently described mucosal route for immunization^71^.

McConnell et al.^71^ compared in mice the oral and colonic routes for vaccination. Using ovalbumin adjuvanted with cholera toxin, they found that colonic administration induces significantly higher levels of colonic and vaginal IgA and serum IgG than the oral route. Based on their findings, they suggested that the colonic route may be an appropriate route for vaccinating against sexually transmitted infections^71^. Draining of the urogenital and the gastrointestinal tracts to the same lymph nodes supports this recommendation^71^. Patient acceptance and easy of use could be improved by oral delivery targeting the colon that protects the vaccine formulation through passage of the upper gastrointestinal tract. Here, we found better protection in mice immunized by the col. + i.m. + s.c. routes than those vaccinated i.n. + i.m. + s.c. supporting the premise that the proximity of the draining lymph nodes from the vaccination site to the portal of infection is important. Also, resident memory T-cells may be more abundant in the genital tract following colonic compared to i.n. immunization^72^.

Individuals get exposed to pathogens at different times following vaccination. Thus, the overall efficacy of a vaccine should be determined over an extended period of time. To assess the cumulative efficacy of the vaccine over the study period, we combined the results of the three challenges at 80, 120, and 180 days postimmunization. The most robust protection was obtained in mice vaccinated by the colonic followed by the i.m./s.c. routes while the weakest protection was observed in the group vaccinated three times i.m./s.c. Mice immunized by the colonic route, followed by i.m./s.c. routes, had robust protection except on D120. We do not have an explanation for this finding since the immunological parameters do not reflect a failure of the vaccination at that particular point. The same result was observed in the primary experiment and the replicate suggesting that it is reproducible. Further work needs to be done to clarify this unexpected result.

Carmichael et al.^27^ were the first to demonstrate the ability of a recombinant chlamydial subunit vaccine to elicit significant protection against vaginal shedding and infertility in the mouse model. These authors tested several routes for immunization including mucosal only, systemic only, or combinations of mucosal, followed by systemic routes. Mice were vaccinated four times at 2 weeks intervals, CpG-1826 plus Montanide ISA 720 (only systemic) were used as adjuvants, and the animals were challenged 28 days after the last immunization. Mice immunized by a combination of mucosal and systemic routes were the best protected while those vaccinated only in the mucosa had minimal protection. Groups immunized i.m. + s.c. four times had significant protection but not as robust as those vaccinated by two mucosal, vaginal + colonic or intranasal + sublingual, followed by i.m. + s.c. routes. Our data confirm the results by Carmichael et al.^27^ in that a combination of mucosal and systemic routes of immunization elicits the most robust protective immune responses. Specifically, here, mice vaccinated by colonic followed by i.m. + s.c. routes were the best protected. Vaccination four times i.m. + s.c., also induced quite robust protective responses. Using only systemic routes will greatly facilitate the delivery of a chlamydial vaccine particularly if it can be administered simultaneously with the HPV vaccine.

The results of these analyses are very encouraging since we observed significant decreases in the number of mice with positive vaginal cultures, the length of time animals had positive vaginal cultures, number of vaginal cultures and the total number of IFU recovered. This indicates that transmission rates will significantly decline. In addition, significant increases in fertility rates and number of embryos were observed in vaccinated individuals compared with their respective negative controls. These increases in fertility are the major target of a chlamydial vaccine. In humans, these vaccine formulations should also protect against PID, chronic abdominal pain, and ectopic pregnancy since they have the same underlying pathogenic mechanisms as infertility.

In conclusion, vaccination of mice by mucosal plus systemic routes, or systemic routes only, with *C. muridarum* MOMP, using a combination of a Th1 and a Th2 adjuvant, can significantly decrease vaginal shedding and protect against long-term sequelae over a third of the life-span of the animals. This suggests, that a Chlamydia vaccine may be able to elicit protection during most of the reproductive life span of human females. To our knowledge this is a first time, that this broad and long-term protection has been achieved with a subunit Chlamydia vaccine.

## Methods

### Stocks of *C. muridarum*

*C. muridarum* (strain Nigg II) was obtained from the American Type Culture Collection (Manassas, VA) and was grown in HeLa-229 cells^73^. Elementary bodies (EB) were purified from *C. muridarum* infected cultures using density gradient centrifugation and were stored at −80 °C until used^24^. The stock of *C. muridarum* was tittered in HeLa-229 cells^39^.

### Preparation of recombinant *C. muridarum* MOMP and *Neisseria gonorrhoeae* Porin B

The cloning, expression and purification of *C. muridarum* MOMP and *N. gonorrhoeae* PorB (*Ng*-PorB) were performed as previously described^74,75^. By the limulus amoebocyte assay (BioWhittaker, Inc., Walkersville, MD), the recombinant proteins had less than 0.05 EU of LPS/mg of protein^75^. Before immunization the antigens were dialyzed against PBS (pH 7.4) with 0.05% of the zwitterionic detergent Z3-14 (Anatrace: Maumee, OH).

### Vaccination protocols

Three-week-old BALB/c (H-2^d^) mice (Charles River Laboratories; Wilmington, MA) were immunized by the i.n., col., i.m., or s.c. routes with MOMP (10 μg/mouse/immunization) as follows^27,71^. One group of mice was vaccinated twice by the col. route followed by two times via the i.m. + s.c. routes. Another group was immunized twice i.n. followed twice i.m. + s.c. A third set of mice was vaccinated three times i.m. + s.c., while a fourth group was immunized four times i.m. + s.c. All immunizations were performed at two-weeks intervals. CpG-1826 (10 µg/mouse/immunization; 5′-TCCATGACGTTCCTGACGTT-3′; Trilink Biotechnologies Inc., San Diego, CA) and Montanide ISA 720 VG (Seppic Inc, Fairfield, NJ; at a 30:70 volume ratio of MOMP plus CpG to Montanide) were used as adjuvants^27,68,75^. Montanide was delivered only by systemic routes. Negative control groups were inoculated with *Ng*-PorB as antigen using the same adjuvants and routes of immunization as the experimental groups. At the time when the other groups received the first immunization, the positive control group was vaccinated once i.n. with 10^4^ inclusion forming units (IFU) of *C. muridarum*. All experiments were replicated. We had complied with all relevant ethical regulations for animal testing and research. All animal protocols were approved by the University of California Irvine, Animal Care and Use Committee.

### Immunoassays

Blood was collected before the genital challenge by periorbital puncture. *C. muridarum* specific antibody titers in serum were determined by an enzyme linked immunosorbent assay (ELISA)^28,39^. In brief, 96-well plates were coated with 100 μl of *C. muridarum* EB in PBS at a concentration of 10 μg of protein/ml. Serum (100 μl) was added to each well in 2-fold serial dilutions. After incubation at 37 °C for 1 h, the serum was discarded and the wells were washed three times with PBS. The plates were incubated with horseradish peroxidase-conjugated goat antimouse IgG1, or IgG2a, antibodies (BD Pharmingen, San Diego, CA). The binding was measured in an EIA reader (Labsystem Multiscan, Helsinki, Finland) using 2′-2′-azinobis (3-ethylbenzthiazoline-6-sulfonic acid) as the substrate. Preimmunization sera were used as negative controls. In vaginal washes, the levels of *C. muridarum* specific IgG and IgA antibodies were determined using the same methodology. The titers are expressed as the reciprocal of the dilution.

To detect antibodies, elicited by vaccination, to linear epitopes of *C. muridarum* MOMP, overlapping 25-mers corresponding to the amino acid sequence of mature MOMP, were chemically synthesized (SynBioSci Corp.; Livermore, CA)^30^. Peptide 25 (p25) overlapped the N-termini and C-termini of MOMP. The peptides were adsorbed onto high binding affinity ELISA plates (1 µg/well of a 96-well plate) and antibody binding was determined in triplicates as described above using anti-mouse IgG^76^.

In vitro neutralization assays were performed as follows^77^. *C. muridarum* (1 × 10^4^ IFU) was added to mouse sera, and two-fold serial dilutions made with Ca^2+^ and Mg^2+^ free PBS, pH 7.2, supplemented with 5% guinea pig serum as a source of complement. Following incubation for 45 min at 37 °C, the mixtures were inoculated by centrifugation into HeLa-229 cells grown on shell vials. After 30 h at 37 °C, the monolayers were fixed and stained with a pool of monoclonal antibodies developed in our laboratories^39^. The titer of a sample was the dilution that yielded 50% neutralization relative to the negative control serum from preimmunized mice.

A T-cell lymphoproliferative assay was performed using T-cells from the spleen^28,39^. Briefly, spleens from each group of mice were collected, teased and enriched for T-cells by passage over a nylon wool column. T-enriched cells (>90%) were counted and 10^5^ cells/well were aliquoted into a 96-well plate. Antigen presenting cells (APC) were prepared by irradiating splenocytes with 3300 rads. UV-inactivated *C. muridarum* EB were added at a concentration of 10 EB to 1 APC. Control wells received medium alone, as negative control, or concanavalin A (ConA; 5 μg/ml), as a positive nonspecific pan-T-cell mitogen. Cell proliferation was measured by addition of 1 μCi of (methyl ^3^H) thymidine per well. The mean counts per minute (cpm) was determined from triplicate cultures. The delta cpm (Δcpm) is the cpm in cultures from T cells stimulated with EB minus the cpm from cultures stimulated with medium. The stimulation index (S.I.) was calculated by dividing the number of cpm in cultures from T-cells stimulated with EB by the number of cpm from cultures stimulated with medium.

### Cytokine measurements

Levels of IFN-γ were determined by EIA using commercial kits following the manufacturer instructions (BD-Pharmingen). T-cells from the spleen were stimulated with EB as described above and IFN-γ was measured in the tissue culture supernatants collected after 48 h of incubation.

### Genital challenge and vaginal cultures

Groups of mice were challenged with 10^5^ IFU of *C. muridarum* in the left ovarian bursa at 60, 120, or 180 days after the last immunization^21,39^. Following the genital challenge vaginal samples were collected with swabs for a period of 6 weeks. The first vaginal culture was collected 7 days after challenge. The specimens were cultured in HeLa-229 cells and *C. muridarum* inclusion forming units (IFU) were stained and counted as described^39^.

### Fertility studies

At 7 weeks following the genital challenge, four females were housed in the same cage with a proven breeder male mouse for 18 days and the body weight was used to monitor pregnancy^21,39^. Mice that did not become pregnant were mated a second time with a male mouse that had fathered during the first mating. After euthanasia of pregnant mice, the number of embryos in each uterine horn was counted and bilateral fertility was defined as at least one embryo/uterine horn.

### Statistical analyses

The Mann–Whitney *U*-test, Fisher’s exact test, and the Student’s *t*-test were used for statistical analysis using the program SigmaStat version 3.5. A *P* value of < 0.05 was considered significant.

### Reporting summary

Further information on research design is available in the Nature Research Reporting Summary linked to this article.

## Supplementary information

Reporting Summary

## Data Availability

The authors confirm that all relevant data are included in the manuscript.
