# Supplementary material for: Vaccination with the recombinant major outer membrane protein elicits long-term protection in mice against vaginal shedding and infertility following a Chlamydia muridarum genital challenge
Source: NPJ Vaccines. 2020 Oct 1;5:90. doi: 10.1038/s41541-020-00239-7 (PMC7530680; doi:10.1038/s41541-020-00239-7)
Supplement: Supplementary file 1 — Reporting Summary [file 41541_2020_239_MOESM1_ESM.pdf]

nature research

Corresponding author(s): Luis M. de la Maza

Last updated by author(s): Aug 19, 2020

Nature Research wishes to improve the reproducibility of the work that we publish. This form provides structure for consistency and transparency in reporting. For further information on Nature Research policies, see our Editorial Policies and the Editorial Policy Checklist.

Statistics

For all statistical analyses, confirm that the following items are present in the figure legend, table legend, main text, or Methods section.

n/a	Confirmed	
☐	☒	The exact sample size (*n*) for each experimental group/condition, given as a discrete number and unit of measurement
☐	☒	A statement on whether measurements were taken from distinct samples or whether the same sample was measured repeatedly
☐	☒	The statistical test(s) used AND whether they are one- or two-sided<br>*Only common tests should be described solely by name; describe more complex techniques in the Methods section.*
☐	☒	A description of all covariates tested
☐	☒	A description of any assumptions or corrections, such as tests of normality and adjustment for multiple comparisons
☐	☒	A full description of the statistical parameters including central tendency (e.g. means) or other basic estimates (e.g. regression coefficient) AND variation (e.g. standard deviation) or associated estimates of uncertainty (e.g. confidence intervals)
☐	☒	For null hypothesis testing, the test statistic (e.g. *F*, *t*, *r*) with confidence intervals, effect sizes, degrees of freedom and *P* value noted<br>*Give P values as exact values whenever suitable.*
☒	☐	For Bayesian analysis, information on the choice of priors and Markov chain Monte Carlo settings
☒	☐	For hierarchical and complex designs, identification of the appropriate level for tests and full reporting of outcomes
☒	☐	Estimates of effect sizes (e.g. Cohen's *d*, Pearson's *r*), indicating how they were calculated

Our web collection on statistics for biologists contains articles on many of the points above.

Policy information about availability of computer code

Data collection: No software codes were used to collect data

Data analysis: Data were analyzed using Prism 6, SigmaStat 3.5, and Excel 2011 softwares

For manuscripts utilizing custom algorithms or software that are central to the research but not yet described in published literature, software must be made available to editors and reviewers. We strongly encourage code deposition in a community repository (e.g. GitHub). See the Nature Research guidelines for submitting code & software for further information.

Data

Policy information about availability of data

All manuscripts must include a data availability statement. This statement should provide the following information, where applicable:

- Accession codes, unique identifiers, or web links for publicly available datasets
- A list of figures that have associated raw data
- A description of any restrictions on data availability

All data supporting the findings are available within the paper.

Please select the one below that is the best fit for your research. If you are not sure, read the appropriate sections before making your selection.

☒ Life sciences ☐ Behavioural & social sciences ☐ Ecological, evolutionary & environmental sciences

For a reference copy of the document with all sections, see nature.com/documents/nr-reporting-summary-flat.pdf

All studies must disclose on these points even when the disclosure is negative.

Sample size: Sample size and power were calculated using SigmaStat software 3.5

Data exclusions: No data were excluded

Replication: Each experiment was repeated at least once.

Randomization: Animals were ramdomly assign to groups

Blinding: Investigators were blinded during data collection and analyses.

We require information from authors about some types of materials, experimental systems and methods used in many studies. Here, indicate whether each material, system or method listed is relevant to your study. If you are not sure if a list item applies to your research, read the appropriate section before selecting a response.

Materials & experimental systems

n/a	Involved in the study	
[ ]	[x]	Antibodies
[ ]	[x]	Eukaryotic cell lines
[x]	[ ]	Palaeontology and archaeology
[ ]	[x]	Animals and other organisms
[x]	[ ]	Human research participants
[x]	[ ]	Clinical data
[x]	[ ]	Dual use research of concern

n/a	Involved in the study	
[x]	[ ]	ChIP-seq
[x]	[ ]	Flow cytometry
[x]	[ ]	MRI-based neuroimaging

Antibodies used: To detect chlamydia inclusions mab 40 to the major outer membrane protein of chlamydia was used, for serum antibody titer goat anti-mouse PIg, IgG, IgG1, IgG2a conjugated to HPR were used as secondary antibodies.

Validation: Goat anti mouse antibodies were purchased from commercial companies and mab 40 was characterized and data were published in Infect Imm. 2008. 76:5581-5587

Policy information about cell lines

Cell line source(s): HeLa 229

Authentication: Purchased from ATCC, USA

Mycoplasma contamination: Cell line was negative for Mycoplasma

Commonly misidentified lines (See ICLAC register): None

Policy information about studies involving animals; ARRIVE guidelines recommended for repo

Laboratory animals: Female and breeder male mice were purchased from Charles River

Wild animals: None

Field-collected samples: None

Ethics oversight: IACUC of University of California, Irvine

Note that full information on the approval of the study protocol must also be provided in the manuscript.
